# A T2* MRI prospective survey on heart iron in thalassemia major patients treated with sequential deferipron-desferrioxamine versus deferasirox

**DOI:** 10.1186/1532-429X-13-S1-P303

**Published:** 2011-02-02

**Authors:** Alessia Pepe, Antonella Meloni, Giuseppe Rossi, Maria Chiara Dell'Amico, Domenico G D'Ascola, Marcello Capra, Aldo Filosa, Paolo Cianciulli, Cristina Salvatori, Gennaro Restaino, Caterina Borgna-Pignatti, Massimo Lombardi

**Affiliations:** 1 “G. Monasterio” Foundation and Institute of Clinical Physiology, CNR, Pisa, Italy; 2 A.O. "Bianchi-Melacrino-Morelli", Reggio Calabria, Italy; 3 Ospedale "G. di Cristina", Palermo, Italy; 4 A.O.R.N. Cardarelli, Napoli, Italy; 5 Ospedale "Sant'Eugenio Papa", Roma, Italy; 6 Università Cattolica del Sacro Cuore, Campobasso, Italy; 7 Università di Ferrara, Ferrara, Italy

## Background

Most deaths in thalassemia major (TM) result from cardiac complications due to iron overload. No data are available in literature about possible different changes in cardiac iron in TM patients treated with sequential deferipron-deferoxamine (DFP-DFO) versus deferasirox (DFX). Magnetic Resonance (MR) is the unique non invasive suitable technique to evaluated quantitatively this issue.

## Aims

Our aim was to prospectively assess the efficacy of the DFP-DFO vs DFX in a cohort of TM patients by quantitative MR.

## Methods

Among the first 739 TM patients enrolled in the MIOT (Myocardial Iron Overload in Thalassemia) network, 253 patients performed a MR follow up study at 18 ± 3 months according to the protocol. We evaluated prospectively the 25 patients treated with DFP-DFO versus the 44 patients treated with DFX between the 2 MR scans. Myocardial iron concentrations were measured by T2* multislice multiecho technique.

## Results

Excellent/good levels of compliance were similar in the 2 groups (DFP-DFO 96% vs DFX 100%; *P*=0.36). At baseline the 2 groups were homogeneous for cardiac iron. Among the patients with no significant myocardial iron overload (MIO) at baseline (global heart T2* ≥ 20 ms), there were no significant differences between groups to maintain the patients without myocardial iron overload (DFP-DFO 95% vs DFX 96%; *P* =1.0). Among the patients with MIO at baseline (global heart T2* < 20 ms), only in the DFX group there was a significant improvement in the global heart T2* value (11 ± 5 ms at baseline versus 16 ± 8 at 18 ± 3 months, *P*=0.0001) and in the number of segment with a normal T2* value (*P*=0.003). The improvement in the global heart T2* was not significantly different in the DFP-DFO versus the DFX group (mean difference global heart T2* 2.2 ± 4.1 ms versus 4.6 ± 4.8 *P*=0.2). Conclusions: Prospectively in the clinical setting over 15 months we did not find significant differences on cardiac iron by quantitative MRI in TM patients treated with sequential DFP-DFO versus the TM patients treated with DFX.

